# The Effects of CO_2_ Additional on Flame Characteristics in the CH_4_/N_2_/O_2_ Counterflow Diffusion Flame

**DOI:** 10.3390/molecules26102905

**Published:** 2021-05-13

**Authors:** Ying Chen, Jingfu Wang, Xiaolei Zhang, Conghao Li

**Affiliations:** 1 MOE Key Laboratory of Enhanced Heat Transfer and Energy Conservation, College of Environmental and Energy Engineering, Beijing University of Technology, Beijing 100124, China; Yingchen0511@outlook.com (Y.C.); zhangxl@emails.bjut.edu.cn (X.Z.); S201905113@emails.bjut.edu.cn (C.L.); 2 Beijing Key Laboratory of Heat Transfer and Energy Conversion, College of Environmental and Energy Engineering, Beijing University of Technology, Beijing 100124, China

**Keywords:** CH_4_ counterflow diffusion flame, CO_2_ dilution, temperature, flame front

## Abstract

The effects (chemical, thermal, transport, and radiative) of CO_2_ added to the fuel side and oxidizer side on the flame temperature and the position of the flame front in a one-dimensional laminar counterflow diffusion flame of methane/N_2_/O_2_ were studied. Overall CO_2_ resulted in a decrease in flame temperature whether on the fuel side or on the oxidizer side, with the negative effect being more obvious on the latter side. The prominent effects of CO_2_ on the flame temperature were derived from its thermal properties on the fuel side and its radiative properties on the oxidizer side. The results also highlighted the differences in the four effects of CO_2_ on the position of the flame front on different sides. In addition, an analysis of OH and H radicals and the heat release rate of the main reactions illustrated how CO_2_ affects the flame temperature.

## 1. Introduction

Natural gas is a relatively clean fuel with the potential to replace traditional coal fuel; it has advantages in adjusting energy structure and controlling pollution control, as well as effectively improving the fuel combustion efficiency [1]. Unfortunately, the main bottleneck problem in the application of natural gas combustion technology is related to NO_X_ emissions. In recent decades, various low-nitrogen natural gas combustion technologies have been proposed to mitigate NO_X_ emissions, among which the most widely used is the exhaust gas recirculation technique (EGR), which mixes an additional diluent (CO, CO_2_, and H_2_O) in the unburned gas mixture or in the oxidizer, thereby reducing gas temperature and NOx formation [2]. In practical applications, the effects of EGR on the performance of the combustion process and its efficiency are similar to dilution combustion [3]. Therefore, dilution combustion has drawn unprecedented attention in recent years since it can be easily and economically implemented. As the main product of hydrocarbon fuel, CO_2_ is usually selected as the diluent for diluted combustion [1,4,5,6,7].

Several researchers have conducted numerous studies on the effect of CO_2_ dilution on different fuels. Xu et al. [8] conducted experiments and simulations to determine the effects of CO_2_ replacement of N_2_ in air on the structure and shape of laminar coflow syngas, with the results showing that CO_2_ addition to the oxidizer decreased the flame temperature, thereby increasing the flame height and radius. Shih et al. [6,9] carried out a numerical study on the effects of CO_2_, H_2_O, and N_2_ additions to a one-dimensional counterflow diffusion flame composed of a H_2_/CO synthetic mixture. The above three dilutants were added to the fuel side. CO_2_ dilution showed the greatest effect in terms of a decrease in flame temperature compared to the other two dilutants, and the large drop in flame temperature resulted from a combined effect of flame radiation and chemical kinetics. Fotache et al. [10] and Jia et al. [7] studied the ignition of CH_4_ diluted with N_2_ and CO_2_ in a counterflow ignition facility, with the results showing that the ignition temperature in CH_4_/N_2_ was lower than that in CH_4_/CO_2_, which was mainly attributed to the higher heat capacity of CO_2_ compared to N_2_. Galmiche et al. [11] investigated the effects of dilutants (CO_2_, H_2_O, and N_2_) on the laminar burning velocities of premixed methane/air combustion through experiments and numerical simulations, while they also analyzed the kinetic and thermal effects of CO_2_ on the laminar burning velocities. The authors proposed an explicit correlation between the heat capacity of the diluent and the laminar burning velocity as a function of the heat capacity possessing a predominant effect on the laminar flame. Jithin et al. [12] and Xie et al. [13] also carried out a series of experimental and numerical studies on the effect of CO_2_ dilution on the laminar burning velocity. Hu et al. [14] conducted numerical studies on the effect of diluents on the laminar burning velocity of premixed methane/air/diluent flames. They categorized the effects of diluents into a dilution effect, thermal diffusion effect, and chemical effect, concluding that, for CO_2_ dilution, the dilution effect had a dominant effect on the laminar burning velocity. The chemical effect of CO_2_ on the laminar flame speeds of O_2_/CH_4_ mixtures was numerically studied in a range of oxygen concentrations and equivalence ratios by Hu et al. [4], with the results showing that the chemical effect on the laminar flame speeds was smaller than the thermal effect, but much bigger than the radiative effect in the calculation domain. They also identified the important reactions involving the third-body efficiency of CO_2_. Yang et al. [15] obtained the chemiluminescent distributions of OH* and CH* in CH_4_/O_2_ diffusion flames diluted with CO_2_. Both Gascoin et al. [16] and Ren et al. [17] investigated the effect of CO_2_ dilutant on NO_X_ emissions in a methane flame. The former authors numerically investigated the quantitative thermal effects of CO_2_, by replacing N_2_ on the oxidizer side, on NOx formation in a counterflow diffusion flame configuration at atmospheric pressure. The latter authors studied the effect of CO_2_ on NO_X_ emission in one-dimensional premixed freely propagating flames. Both sets of results showed a significant reduction in NO emissions due to the CO_2_ dilutant. Wang et al. carried out a numerical study on the radiation reabsorption of CO_2_ on NO formation in CH_4_/air counterflow diffusion flames [18] and premixed flames [19]. Various other studies [20,21,22,23,24] investigated the effects of CO_2_ on soot or NO_X_ emissions in different fuels. Moreover, some researchers studying CO_2_ dilution combustion focused on the influence of CO_2_ on other characteristics of the gas flame, such as the adiabatic flame temperature (AFT) [17,25], extinction limits [6,26,27,28], flame instability [29,30,31,32,33], H_2_ intermediate formation [34,35], and CO concentration [36] using various fuels.

According to the literature review, most studies in the field of CO_2_ dilution only focused on the synergistic effects of CO_2_ on the flame structure, flame characteristic parameters, and NO_X_ emissions in a premixed flame or diffusion flame. Knowledge of the effects of CO_2_ dilution on the flame can be categorized into four aspects, chemical effect, thermal effect, transport effect, and radiative effect, due to the special properties of CO_2_. However, few studies [4,14] placed emphasis on a comparison of these four effects of CO_2_, especially with respect to the diffusion flame. In this regard, CO_2_ can not only dilute the oxidizer side [9] but also the fuel side [8]. Systematic investigations and comparisons of CO_2_ dilution of the oxidizer and fuel sides as a function of these four effects on the diffusion flame are still lacking. Moreover, far too little attention has been paid to the isolated effects of these four properties of CO_2_ dilution on the oxidizer and fuel sides. Previous studies [6,8,9] revealed that the CO_2_ dilutant can reduce the flame temperature; however, the authors did not demonstrate how the CO_2_ reduces the flame temperature. The above review highlights the importance and significance of the current research. Exploring the various uncoupled effects of CO_2_ provides a theoretical basis for in-depth exploration of the overall nature effect of CO_2_. Determining the uncoupled effects of CO_2_ provides important theoretical basis and technical support for the application and development of flue gas recycling technology.

In light of the discussion above, the present study carried out a series of numerical simulations describing one-dimensional counterflow diffusion flames, with the following specific objectives: (1) to isolate and quantify the four effects (chemical effect, thermal effect, transport effect, and radiative effect) of CO_2_ dilution on the flame temperature and the position of the flame front; (2) to compare the four effects of CO_2_ dilution on the flame temperature and the flame front on the fuel side and oxidizer side; (3) to determine the differences in the contribution of these four effects; (4) to reveal how CO_2_ affects the flame temperature in terms of the OH and H radicals, as well as the heat release rate (HRR) of the main reactions.

## 2. Numerical Method

**Figure 1:**
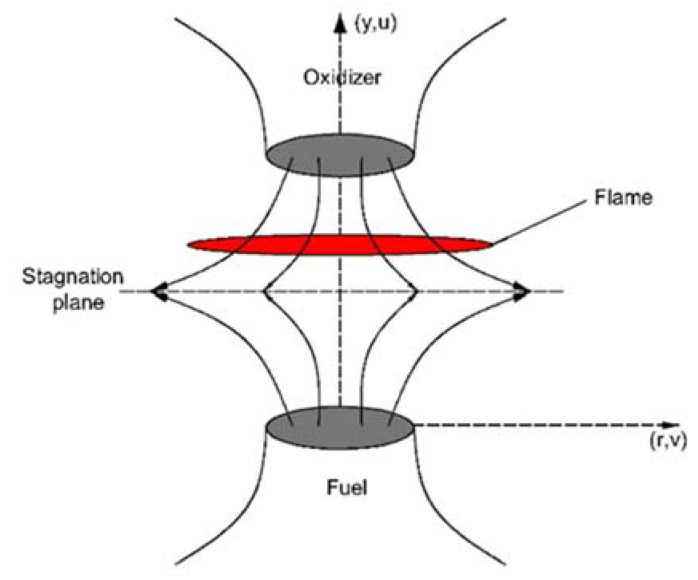
Configuration of the counterflow flame.

An axisymmetric, counterflow laminar diffusion flame with two opposite coaxial jets in models of a fuel and oxidizer was applied in this study. The configuration of the counterflow flame is displayed in Figure 1. The flame is stabilized near the stagnation plane of two opposing jet flows. By assuming the stagnation point flow approximation [37,38], the flame and the flow can be considered as being one-dimensional. A complete mathematical description of this numerical model (including mass, momentum, energy, and composition equations, as well as boundary conditions on the fuel side and oxidizer side) can be found in [39]. The OPPDIF [39] code was used in the current study to solve the mathematical equations of the counterflow diffusion flame. The convection term adopted the upwind difference scheme, and the multicomponent diffusion and thermal diffusion models were used to calculate the diffusion coefficient. An adaptive refinement grid was applied in the calculation process, and the damped Newton method was used to solve the equation. The relative and absolute errors of the iteration process were less than 10^−6^.

For the radiative heat loss ${\dot{q}}_{r}$ in the energy equation, the optically thin model (OTM) was applied to calculate the heat loss contributed by CO, CO_2_, H_2_O, and CH_4_. The corresponding equations describing the radiative effect on the basis of the optically thin approximation are as follows:

(1) $${\dot{q}}_{r}=-{\;4\sigma K}_{P}\left(T^{4}-T_{\infty }{}^{4}\right)$$

(2) $$K_{P}=P\sum^{\;}x_{i}K_{p,i}$$

where *σ* is the Stefan–Boltzmann constant, and $K_{P}$ is the Planck mean absorption coefficient of the flame mixture. *T* and $T_{\infty }$ denote the local and environmental temperature, respectively. In Equation (2), P is the total pressure, whereas $K_{P,i}$ and${\;x}_{i}$ denote, respectively, the mean absorption coefficient and mole fraction of the absorbing and emitting species.

In order to ensure that the stagnation surface of the counterflow flame does not deviate from the center of the fuel and oxidizer nozzles, the calculation limited the two opposing gases to satisfy the following momentum balance equation [20]:

(3) $$\rho_{o}V_{o}^{2}{=\rho }_{F}V_{F}^{2}$$

The global strain rate *α*_*s* was defined as follows [40]:

(4) $$\alpha_{s\;}=\frac{2(-V_{O})}{L}\left[1+\frac{V_{F}}{\left(-V_{O}\right)}\sqrt{\frac{\rho_{F}}{\rho_{O}}}\right]$$

where the subscripts *O* and *F* represent the oxidizer and fuel flow streams, respectively. *ρ* and *V* denote the density and the velocity of the flow streams. *L* is the distance between the fuel and oxidizer nozzles. In Equations (3) and (4), when only considering the absolute values, the expressions describing the velocity on both sides can be expressed as follows:

(5) $$\left|V_{F}\right|=\left|V_{o}\right|\sqrt{\frac{\rho_{o}}{\rho_{F}}}$$

(6) $$\left|V_{O}\right|=\frac{L}{4}{\;\alpha }_{S}$$

In the present study, the distance between the fuel and oxidizer nozzles *L* was specified as 4 cm, the inlet temperature of both the fuel and the oxidizer was specified as 300 K, the strain rate $\alpha_{S}\;$was set to 10 s^−1^, and the pressure was 0.1 MPa. The velocities of the oxidizer side were maintained at a constant value of 0.1 m/s, allowing the velocities of the fuel side to be obtained according to Equations (3)–(6) on the basis of the composition of the fuel and oxidizer.

The purpose of this study was to compare the influence of CO_2_ dilution on the fuel and oxidizer sides by decoupling the thermal, chemical, transport, and radiative properties of CO_2_. Several pairs of artificial and chemically inert species were introduced to isolate the effect of CO_2_: FCO_2_, TCO_2_, XCO_2_, and RCO_2_ as shown in Table 1. The method used to dissociate the effects of CO_2_ was explained as followed:

1. The chemical effect. FCO_2_, which has the same thermal, transport, and radiative properties as CO_2_, but does not participate in the chemical reaction, was introduced to evaluate the chemical effect of CO_2_. Therefore, any differences in the results between FCO_2_ and CO_2_ were entirely contributed by the chemical effect of CO_2_.
2. The thermal effect. TCO_2_, which has the chemical, transport, and radiative properties as FCO_2_, but owns the same thermal properties as normal N_2_, was employed. Thus, any differences in the results between FCO_2_ and TCO_2_ were totally caused by the thermal effect of CO_2_.
3. The transport effect. XCO_2_, which has the same chemical, thermal, and transport properties as FCO_2_, but owns the same transport properties as N_2_. The transport effect of CO_2_ was revealed by the difference in results between FCO_2_ and XCO_2_.
4. The radiative effect. RCO_2_, which has the same chemical, thermal, and transport properties as FCO_2_, but is hot transparent body and cannot contribute to radiative heat transfer. Therefore, the radiative effects of CO_2_ were determined as a function of the difference in results between FCO_2_ and RCO_2_.

As is known, the thermodynamic properties of a gas specie are mainly derived from its enthalpy, entropy, and specific heat capacity. These properties were obtained from polynomial fitting parameters provided in the THERMDAT file. On the other hand, the transport properties of a gas specie are derived from its viscosity, thermal conductivity, and thermal diffusion coefficient, which were calculated as a function of the parameters provided in the TRANDAT file. As shown in Table 1, the TCO_2_ features the same thermodynamic properties as N_2_; thus, the same polynomial fitting parameters were adopted to calculate their thermodynamic coefficient. This was achieved by modifying the TCO_2_ raw input in the THERMDAT file. XCO_2_, with the same transport properties as N_2_, could also be obtained by modifying the XCO_2_ raw input in the THERMDAT file.

The mole fraction of CO_2_ was set to 30% on both the fuel side and the oxidizer side, as the aim of the present study was to compare the difference in CO_2_ effects on each side of the counterflow flame, as opposed to the dilution ratio. The numerically simulated conditions taken into account in this study are summarized in Table 2. GRI-Mech 3.0 was implemented to carry out the simulations in this study, as it is the most popular approach used to characterize CH_4_ and has been validated by numerous research studies.

## 3. Results and Discussion

### 3.1. The CO_2_ Effect on the Flame Temperature

**Figure 2:**
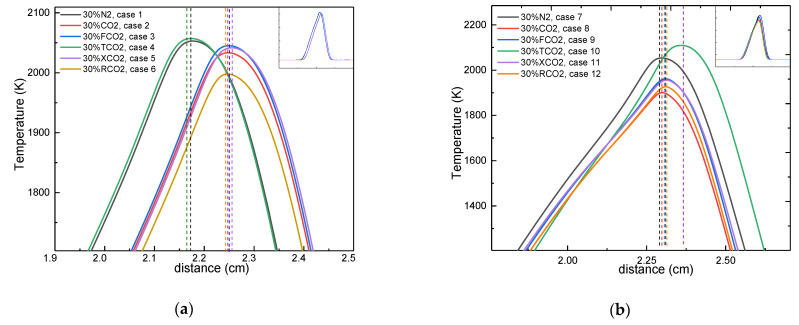
The temperature profile of the counterflow flame upon dilution of the two sides. (**a**)-Left fuel side; (**b**)-Right oxidizer side.

Figure 2 depicts the flame temperature profile when diluted with CO_2_ and various other artificial species on both sides of the counterflow diffusion flame. The dashed line represents the position of the flame front. As clearly seen in this figure, the flame temperature increased and then decreased with distance from the fuel side, reaching its maximum at the flame front (peak value temperature). By comparing cases 1 and 2 and cases 7 and 8, it can be seen that the maximum temperature was decreased due to CO_2_ dilution, whether on the fuel side or the oxidizer side. Additionally, there was an obvious difference in the shift of the flame front position upon dilution of the two sides, with the flame front offset toward the side which was not diluted [16]. Regardless of whether CO_2_ was added to the fuel side or oxidizer side, the maximum flame temperature always resided on the oxidizer side of the configuration, which is consistent with the findings in [42].

**Figure 3:**
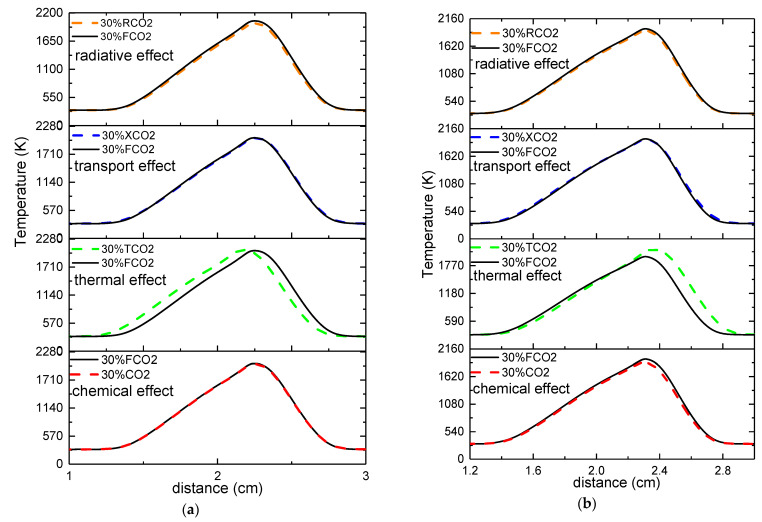
The four effects of CO_2_ on the counterflow flame temperature upon dilution of the two sides. (**a**)-Left fuel side; (**b**)-Right oxidizer side.

Figure 2 also shows that the chemical properties, thermal properties, transport properties, and radiative properties of CO_2_ had an effect on the flame temperature distribution whether it was diluted on the fuel side or the oxidizer side. Figure 3, therefore, individually presents these four effects of CO_2_. When the fuel side was diluted, the effects due to the chemical and transport properties could be neglected. However, the thermal properties and the radiative properties of CO_2_ had a different influence on the flame temperature and the flame front. The thermal properties decreased the flame temperature ahead of the flame front, whereas it increased the flame temperature behind the flame front. The radiative properties of CO_2_ resulted in a slight overall decrease in flame temperature distribution. When the oxidizer side was diluted, no significant changes in temperature distribution occurred as a result of the transport properties of CO_2_, whereas the overall flame temperature distributions were slightly lower due to the chemical and radiative properties of CO_2_. Lastly, the thermal properties had a different influence on the flame temperature distribution on either side of the flame front: a bit higher than the undiluted gas mixture ahead of the flame front but apparently lower than the undiluted gas mixture behind the flame front. It is necessary to note that “no significant change” does not mean that “no change occurred”; a detailed analysis of the four effects is presented later.

In summary, CO_2_ affected the flame temperature distribution as a function of the side which was diluted. A comparison of the four properties allowed distinguishing their primary and secondary effects on the counterflow diffusion flame.

Before analyzing and comparing the effects of the chemical, thermal, transport, and radiative properties of CO_2_ on the flame temperature peak value, their contributions were individually defined as follows:

(7) $$∆T_{\mathrm{Chem}}^{\;\max}{=T}_{\max}\left[{\mathrm{CO}}_{2}\right]-T_{\max}\left[{\mathrm{FCO}}_{2}\right]$$

(8) $$∆T_{\mathrm{therm}}^{\;\max}{=T}_{\max}\left[{\mathrm{FCO}}_{2}\right]-T_{\max}\left[{\mathrm{TCO}}_{2}\right]$$

(9) $$∆T_{Trans\;\;}^{\;\max}{=T}_{\max}\left[{\mathrm{FCO}}_{2}\right]-T_{\max}\left[{\mathrm{XCO}}_{2}\right]$$

(10) $$∆T_{\mathrm{Radia}}^{\;\max}{=T}_{\max}\left[{\mathrm{FCO}}_{2}\right]-T_{\max}\left[{\mathrm{RCO}}_{2}\right]$$

where a positive $∆{T\;}^{\max}$ represents an increase in flame temperature peak value, while a negative $∆T^{\;\max}$ denotes a decrease.

Similarly, the shift in position of the flame front as a function of the four properties of CO_2_ was described using similar equations.

**Figure 4:**
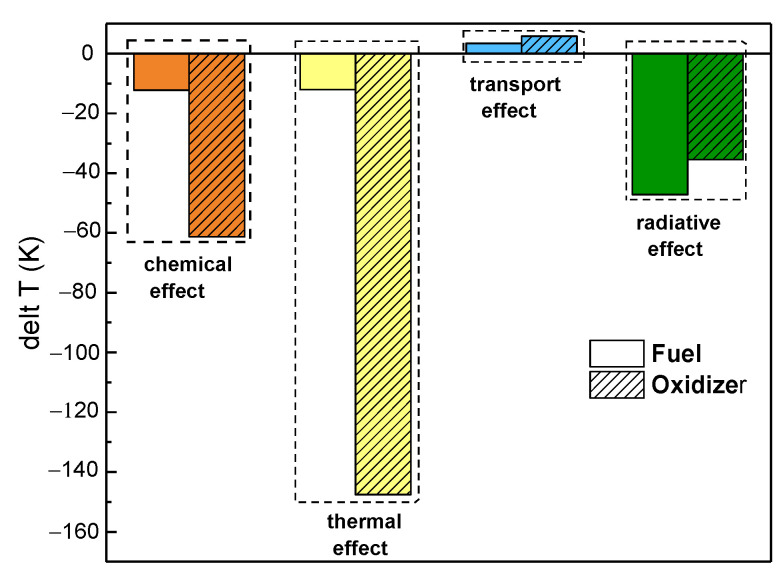
The four effects of CO_2_ on the maximum counterflow flame temperature upon dilution of the two sides.

Figure 4 shows a comparison of the four effects on the maximum flame temperature, where plain and patterned columns represent the change in maximum flame temperature on the fuel side and oxidizer side, respectively. In this figure, it can be seen that, whether on the fuel side or oxidizer side, the chemical properties, thermal properties, and radiative properties decreased the maximum flame temperature, whereas the transport properties increased the maximum flame temperature. Furthermore, on the fuel side with 30% CO_2_ dilution, the radiative properties had the primary negative effect, while the chemical and thermal properties had a roughly equivalent effect. The transport properties had the smallest influence on the maximum flame temperature. On the oxidizer side, with 30% CO_2_ dilution, the thermal properties had the most prominent effect on the maximum flame temperature, whereas the chemical and radiative properties effects contributed approximately one-half and one-third the effect of the thermal properties effect, respectively. Once again, the transport properties had the smallest influence on the maximum flame temperature. Lastly, the effect of the chemical, thermal, and transport properties on the maximum flame temperature was greater on the oxidizer side than the fuel side under 30% CO_2_ dilution. However, the effect of the radiative properties was slightly lower on the oxidizer side.

**Figure 5:**
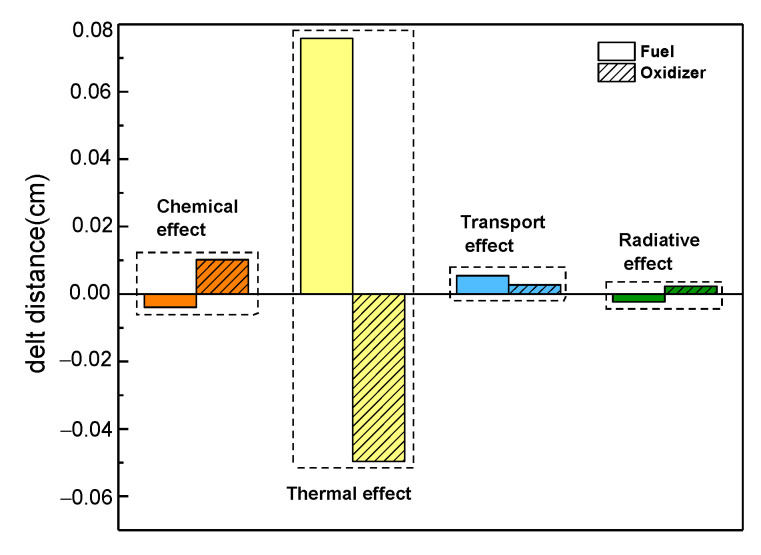
The four effects of CO_2_ on the position of the counterflow flame front upon dilution of the two sides.

Figure 5 presents a comparison of the chemical, thermal, transport, and radiative effects on the position of the flame front under the same CO_2_ dilution ratio. The legends of Figure 5 are the same as those in Figure 4. A positive value represents a deviation of the flame front position toward the oxidizer side, while a negative value represents a deviation toward the fuel side. It can be seen in Figure 5 that the effects of CO_2_ on the position of the flame front differed as a function of which side was diluted. When the fuel side was diluted with CO_2_, the chemical and radiative effects moved the position of the flame front toward the fuel side, whereas the thermal and transport effects moved the position of the flame front toward the oxidizer side. However, when the oxidizer side was diluted with CO_2_, the chemical, transport, and radiative effects moved the position of the flame front toward the oxidizer side, with the opposite outcome seen due to the thermal effect. Consistently, the thermal effect on the position of the flame front was the most conspicuous regardless of which side was diluted.

### 3.2. The CO_2_ Effect on the H and OH Radicals

H and OH radicals are very important for the continuation of chain reactions; moreover, the first step in a combustion reaction starts with the fuel being attacked by free radicals such as OH and H [43], whereby their mole fraction can also indicate the combustion intensity.

**Figure 6:**
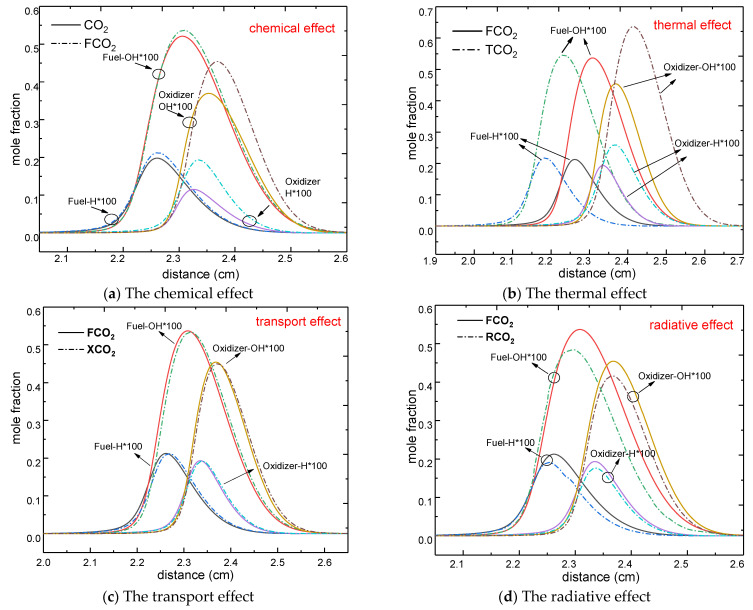
Comparison of the four effects on the distribution of H and OH radicals in the counterflow diffusion flame on the fuel side and oxidizer side (**a**)-Chemical effect; (**b**)-Thermal effect; (**c**)-Transport effect; (**d**)-Radiative effect.

As discussed above, CO_2_ had a strong influence on the flame temperature and the position of the flame front, as well as on the reaction zone (the thickness of the flame). The distributions of intermediate species and highly active radicals of combustion are associated with the flame temperature and the reaction zone. To illustrate the influence of the chemical, thermal, transport, and radiative properties of CO_2_ on the two most important highly active radicals (H and OH), a comparison of their effects on the mole fraction of H and OH radicals is depicted in Figure 6. As expected, the obtained profiles were consistent with the temperature distribution (Figure 3 and Figure 4), i.e., an initial increase followed by a decrease, with the maximum value of the mole fraction of H and OH radicals being near the flame front. Taking the CO_2_ dilution on the fuel side as an example without considering the four effects on the mole fraction of H and OH radicals, the maximum H and OH mole fractions were located at 2.26 and 2.31 cm, respectively, while the flame front was located at 2.24 cm.

**Figure 7:**
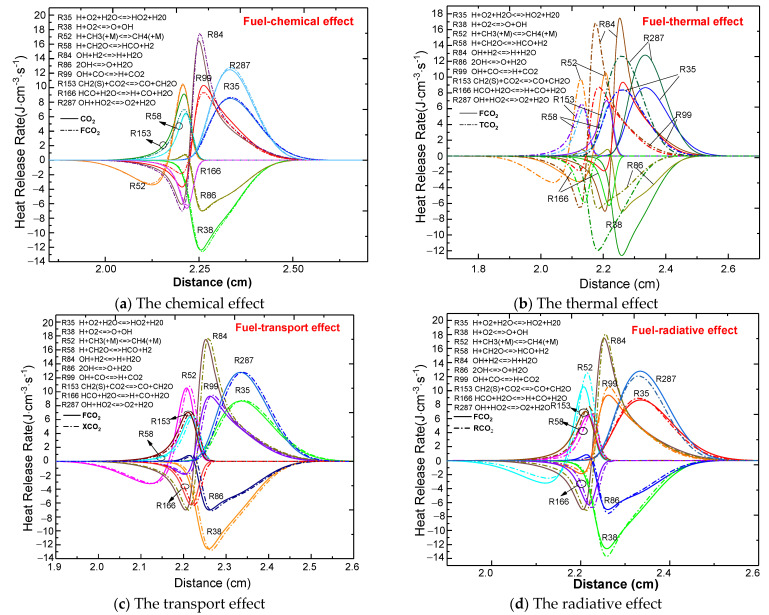
The heat release rate (HRR) profiles of the counterflow diffusion flame as a function of the four effects of CO_2_ on the fuel side (**a**)-Chemical effect; (**b**)-Thermal effect; (**c**)-Transport effect; (**d**)-Radiative effect.

The previous temperature curves allow for analysis and understanding of the changes in H and OH radicals under the influence of the four properties of CO_2_. As shown in Figure 6a,d, both the chemical and the radiative properties of CO_2_ lowered the H and OH mole fractions regardless of the side which was diluted; however, their peak positions showed no significant change. Similarly, the thermal properties of CO_2_ (Figure 7b) significantly decreased the H and OH mole fractions upon dilution of the oxidizer side, whereas a weaker effect was identified when CO_2_ was added to the fuel side. Furthermore, the thermal properties of CO_2_ also had an effect on the peak position of H and OH radicals, which notably moved toward the side which was not diluted. The transport effects (derived from the difference between FCO_2_ and XCO_2_) contributed least to the overall distribution of the mole fraction and peak position of H and OH radicals.

In summary, CO_2_ dilution on either side of the counterflow diffusion flame led to a decrease in the concentration of H and OH radicals and, thus, a reduction in combustion intensity. Moreover, the peak position of free radicals represents the combustion area with the highest intensity, which moves toward the center of the domain. The analyses of the mole fraction of H and OH radicals were consistent with the change in temperature distribution as a function of CO_2_ dilution.

The origin of the effects of CO_2_ dilution on the temperature distribution can be attributed to the behavior of the combustion heat release rate of the involved reactions. To clearly elucidate the effect of the addition of CO_2_ to different sides of the counterflow diffusion flame on its temperature, a heat release rate analysis was carried out. The top ten elementary reactions with the largest heat release rate were selected to show the uncoupled effects of CO_2_ on the HRR.

### 3.3. The CO_2_ Effect on the Heat Release Rate (HRR)

#### 3.3.1. Comparison of the Four Effects upon Dilution of the Fuel Side

Wei et al. [44] pointed out that the addition of CO_2_ to the CH_4_ fuel side will reduce the global heat release rate significantly. The four effects of CO_2_ on the profiles of heat release rate are plotted in Figure 7. As shown in Figure 7a, before the flame front (2.23 cm), the chemical properties of CO_2_ had a great effect on R84 (positive effect: less heat absorbed), R99 (negative effect: more heat absorbed), and R153 (positive effect: more heat released). However, it can be clearly seen that the negative impact was greater than the two positive impacts at the same position. Behind the flame front, the chemical properties of CO_2_ also had an effect on R35 (negative effect: more heat absorbed), R84 (negative effect: more heat absorbed), R287 (negative effect: more heat absorbed), and R99 (positive effect: more heat released). Similarly, the negative influence was stronger than the positive influence at the same position. Therefore, the synergistic influence of the chemical properties of CO_2_ on the heat release rate was negative, thereby leading to a decrease in temperature. The effects of the thermal, transport, and radiative properties on HRR are depicted in Figure 7b–d, respectively, and their influence can be analyzed in a similar manner. It is worth noting that the chemical, transport, and radiative properties of CO_2_ declined or improved the HRR at the same position for the target elementary reaction, as well as decreased or increased the peak value. However, the thermal properties did not affect the peak value of HRR for each target elementary reaction. As shown in Figure 4, the distribution of the flame temperature with FCO_2_ dilution (considering the thermal properties of CO_2_) moved toward the oxidizer side compared with TCO_2_ dilution, which can be explained by the change in distribution of the HRR.

#### 3.3.2. Comparison of the Four Effects upon Dilution of the Oxidizer Side

**Figure 8:**
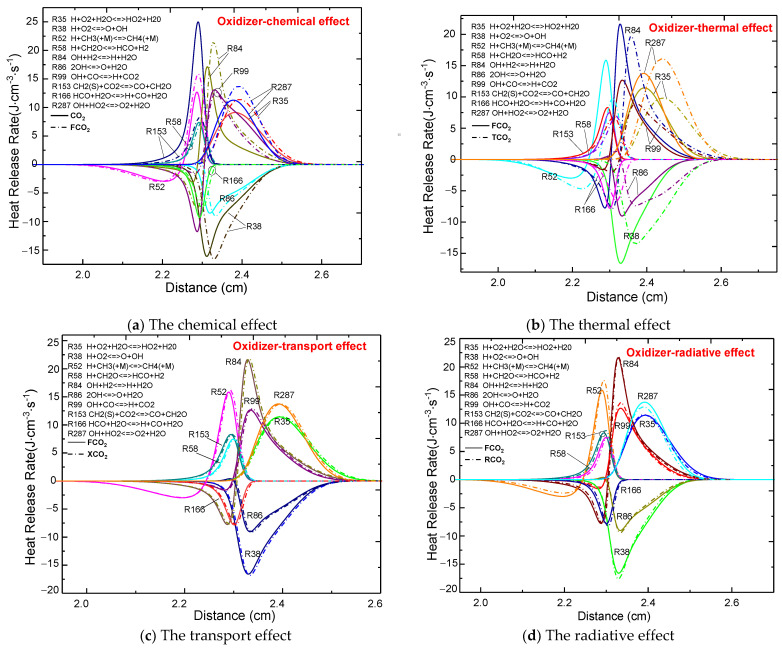
The HRR profiles of the counterflow diffusion flame as a function of the four effects of CO_2_ on the oxidizer side (**a**)-Chemical effect; (**b**)-Thermal effect; (**c**)-Transport effect; (**d**)-Radiative effect.

The effects of the four properties of CO_2_ on the HRR when the oxidizer side was diluted were also investigated. As shown in Figure 8, both the chemical and the thermal properties of CO_2_ moved the HRR distribution of the main target elementary reaction toward the fuel side, leading to a change in the HRR peaks of the exothermic and endothermic elementary reactions. On the other hand, the transport and radiative properties only affected the HRR peak of each elementary reaction, with no obvious effect on its distribution. When comparing FCO_2_ (without a chemical effect) with CO_2_, a decrease occurred in the HRR of the exothermic reactions R35, R84, R287, and R52 involving H and OH radicals. Figure 6a previously presented the distribution of H and OH radicals with CO_2_ and FCO_2_, whereby a decrease in their molar fraction inhibited the relevant elementary reactions, thus resulting in a reduction in the heat release rate. Furthermore, a noticeable thermal effect was reflected in the position of the flame front in Figure 5. The positions of the flame front with FCO_2_ (with thermal properties) and TCO_2_ (without thermal properties) were at 2.3 cm and 2.36 cm, respectively. According to Figure 8b, the overall distribution of the FCO_2_ HRR was shifted toward the fuel side compared with TCO_2_.

## 4. Conclusions

In this paper, a numerical study of CO_2_ dilution on different sides of a laminar counterflow diffusion flame was conducted using the OPPDIF code of ANSYS CHEMKIN-PRO. In order to isolate the CO_2_ effects on the flame temperature, the position of the flame front, the H and OH mole fractions, and the heat release rate, the authors introduced four pairs of artificial CO_2_ species to uncouple the chemical, thermal, transport, and radiative effects of CO_2_ on the above characteristics of the flame. The numerical results demonstrated that the effects of the four properties of CO_2_ were often contrasting, as summarized below.

1. The synergistic effects of CO_2_ on the flame temperature were negative regardless of which side was diluted, with a greater effect seen upon dilution of the oxidizer side. The chemical, thermal, and radiative properties led to a decrease in flame temperature, whereas the transport properties increased the flame temperature. Moreover, the thermal properties had a more prominent effect on the flame temperature upon dilution of the fuel side, whereas the radiative properties had a predominant effect on the flame temperature upon dilution of the oxidizer side.
2. The effects of CO_2_ showed great differences with respect to the position of the flame front. When the fuel side was diluted with CO_2_, the chemical and radiative effects moved the position of the flame front toward the fuel side, whereas the thermal and transport effects moved the position of the flame front toward the oxidizer side. However, when the oxidizer side was diluted with CO_2_, the chemical, transport, and radiative effects moved the position of the flame front toward the oxidizer side, with the opposite outcome seen due to the thermal effect. Consistently, the thermal effect on the position of the flame front was the most conspicuous regardless of which side was diluted.
3. The chemical and radiative effects of CO_2_ resulted in a slight decrease in the mole fraction of H and OH radicals regardless of which side was diluted. The thermal properties of CO_2_ only affected the distribution of H and OH radicals upon dilution of the fuel side, with no significant effect on its peak. However, the thermal properties of CO_2_ not only affected the distribution of OH and H radicals upon dilution of the oxidizer side, but also reduced their maximum mole fraction, whereas the effect of transport properties on OH and H radicals could be neglected.
4. The influence of CO_2_ on the heat release rate was consistent with its influence on temperature and free radical concentration regardless of which side was diluted.

## Figures and Tables

**Table 1:** The specific properties of the artificial species [41].

	Chemical	Thermal	Transport	Radiative
CO_2_	√	√	√	√
FCO_2_	—	√	√	√
TCO_2_	—	N_2_	√	√
XCO_2_	—	√	N_2_	√
RCO_2_	—	√	√	—

**Table 2:** The conditions for each case of CO_2_ added to both sides of the counterflow flame.

**Flame** **Case**	**Strain Rate (s^−1^)**	**Distance** **(m)**	**Fuel** **Composition** **(Mole Fraction)**	**Oxidizer** **Composition** **(Mole Fraction)**	$V_{O}\;(m/s)$	$V_{F}\;(m/s)$
1	10	0.04	70% CH_4_ + 30% N_2_	79% N_2_ + 21% O_2_	0.10	0.1309
2	10	0.04	70% CH_4_ + 30% CO_2_	79% N_2_ + 21% O_2_	0.10	0.1087
3	10	0.04	70% CH_4_ + 30% FCO_2_	79% N_2_ + 21% O_2_	0.10	0.1087
4	10	0.04	70% CH_4_ + 30% TCO_2_	79% N_2_ + 21% O_2_	0.10	0.1087
5	10	0.04	70% CH_4_ + 30% XCO_2_	79% N_2_ + 21% O_2_	0.10	0.1087
6	10	0.04	70% CH_4_ + 30% RCO_2_	79% N_2_ + 21% O_2_	0.10	0.1087
7	10	0.04	100% CH_4_	79% N_2_ + 21% O_2_	0.10	0.1341
8	10	0.04	100% CH_4_	30% CO_2_ + 49% N_2_ + 21% O_2_	0.10	0.1449
9	10	0.04	100% CH_4_	30% FCO_2_ + 49% N_2_ + 21% O_2_	0.10	0.1449
10	10	0.04	100% CH_4_	30% TCO_2_ + 49% N_2_ + 21% O_2_	0.10	0.1449
11	10	0.04	100% CH_4_	30% XCO_2_ + 49% N_2_ + 21% O_2_	0.10	0.1449
12	10	0.04	100% CH_4_	30% RCO_2_ + 49% N_2_ + 21% O_2_	0.10	0.1449
